# Sexual Function in Women with Polycystic Ovary Syndrome Living in Stable Heterosexual Relationships: A Cross-Sectional Study

**DOI:** 10.3390/jcm13082227

**Published:** 2024-04-12

**Authors:** Anna Warchala, Paweł Madej, Marta Kochanowicz, Marek Krzystanek

**Affiliations:** 1 Department and Clinic of Psychiatric Rehabilitation, Faculty of Medical Sciences, Medical University of Silesia in Katowice, Ziołowa 45/47, 40-635 Katowice, Poland; awarchala@sum.edu.pl; 2 Department of Gynecological Endocrinology, Faculty of Health Science in Katowice, Medyków 14, Medical University of Silesia, 40-752 Katowice, Poland; 3 Clinical Department of Obstetrics, Gynecology and Gynecological Oncology in Kędzierzyn-Koźle, Roosvelta Str. 2, 47-200 Kędzierzyn-Koźle, Poland; marthakoch@interia.pl

**Keywords:** polycystic ovary syndrome, female sexual function, arousal, desire, sexual functioning of the couple

## Abstract

**Background/Objective:** The prevalence and character of female sexual dysfunction (FSD) in polycystic ovary syndrome (PCOS) have not been precisely determined. The aim of this study was to assess FSD using the Changes in Sexual Functioning Questionnaire (CSFQ-14) in women with PCOS and their partners compared to a control group, as well as correlations between five subscales, the total score of the CSFQ, and seven questions of the Visual Analogue Scale (VAS). **Methods:** The study sample (*N* = 160) comprised two groups: (1) women with PCOS and their partners (*n* = 91) and (2) women without PCOS and their partners (control group; *n* = 69). **Results:** The total scores of the CSFQ did not reveal FSD in either group of women. Regarding all subscales and the total score, the analysis showed a statistically significant difference between women and their partners (in all cases: *p* < 0.001). The discrepancy in arousal between women and men in the PCOS group was large (the mean difference was −2.32; *t* = −11.29, *p* < 0.001, Cohen’s d = −1.26). The importance (VAS1), the level (VAS7) of sexual satisfaction, and the intensity of sexual thoughts (VAS2) correlated with almost all domains of the CSFQ. **Conclusions:** In conclusion, normal sexual function in PCOS does not mean proper sexual functioning in a sexual relationship.

## 1. Introduction

Polycystic ovary syndrome (PCOS) affects 5–18% of women and is a complex condition with impacts across the lifespan [1]. It is recommended to base its diagnosis on the 2003 Rotterdam criteria and confirm with two of the three criteria: hyperandrogenism, irregular cycles, and polycystic ovary morphology [2]. The diagnostic criteria generate four phenotypes, and the clinical features are heterogeneous. This is reflected in the fact that PCOS remains the most common endocrine disease of women of reproductive age [3], the most common cause of anovulatory infertility [4], and a substantial contributor to an early onset of type 2 diabetes [5], as well as psychological disorders [6]. 

Numerous clinical studies have provided a greater understanding of the substantial psychological features associated with PCOS. It is reported that increased prevalences of depression, anxiety, negative body image, low self-esteem, and psychosexual dysfunction are associated with high levels of distress and adverse effects on the quality of life in women with PCOS [7,8]. Quality of life scores are often reduced in women with PCOS, with concerns around weight and infertility having the most detrimental impact [6]. However, obesity, hyperandrogenism, and infertility are weakly associated with symptoms of depression and anxiety scores [6]. As it was shown in a 2019 community-based study, women with PCOS also have an increased prevalence of disordered eating, such as binge eating disorder, compared to controls [9].

Female sexual dysfunction (FSD) is defined in the fifth edition of the Diagnostic and Statistical Manual of Mental Disorders (DSM-5) as a frequent, long-lasting, and distressing problem concerning desire, arousal, orgasm, or pain and is associated with various conditions characterized by negative mental and physical outcomes [10]. Despite specific studies, the prevalence and character of FSD in PCOS have not been precisely determined, and the conclusions from these studies are inconsistent [11,12,13]. The gaps in knowledge make the condition interesting for further study. Establishing a diagnosis for sexual problems in patients with PCOS might give rise to clinical trials aimed at improving sexual disturbances in this group of patients.

## 2. Materials and Methods

The aim of our study was to assess sexual function in women with PCOS and their partners compared to a similar control group. We decided to use the Changes in Sexual Functioning Questionnaire (CSFQ) because it allowed us to assess sexual functions within the same domains in women and men. We wanted to examine sexual function in women with PCOS in the context of their partners. We also tried to explore the impact of self-perception in the areas of sexual satisfaction, personal attractiveness, excessive hair, social struggles, intensity of sexual thoughts and fantasies, and painful sexual encounters on specific domains in CSFQ. We decided to put forward four hypotheses. (1) The importance and level of sexual satisfaction correlates positively with specific dimensions of the CSFQ. (2) The perceived level of personal attractiveness correlates positively with specific dimensions of the CSFQ and the perceived impact of excessive hair on personal sexuality and social struggles due to appearance correlating negatively with specific dimensions of the CSFQ. (3) The intensity of sexual thoughts and fantasies correlates positively with specific dimensions of the CSFQ. (4) The occurrence of painful sexual encounters correlates negatively with specific dimensions of the CSFQ. Additionally, we analyzed demographic and physical correlates of the sexual functioning of women with PCOS. This study was approved by the Ethics Committee of the Medical University of Silesia, No.: PCN/CBN/0022/KB1/77/21. 

The inclusion criteria comprised women diagnosed with polycystic ovary syndrome (PCOS) according to the Rotterdam criteria, aged 18–40, with partners, and who gave written consent. The control group included women without PCOS, aged 18–40, with their partners, and who gave written consent. 

The patients with PCOS were recruited through the Department of Gynecological Endocrinology of Medical University of Silesia in Katowice (Poland). The participants of the control group were recruited from the medical students of the Medical University of Silesia and the medical staff at the Upper Silesian Medical Centre in Katowice.

Patients with PCOS were recruited to the study group during their first diagnostic hospitalization at the Department of Gynecological Endocrinology. The diagnosis of PCOS was confirmed by gynecologists and endocrinologists according to the Rotterdam criteria and based on a physical examination and a set of laboratory tests tailored to each patient’s symptoms. The topic of sexuality was not mentioned prior to the patients taking the survey. The information provided to the respondents included a brief description of the significance of the sexual function in women with PCOS for their further life satisfaction. Participating patients with a stable male partner gave written consent to the proposed study and completed a set of questionnaires for women (demographic, CSFQ-F-C, VAS) at the end of their stay in the hospital ward. The questionnaire intended for men (CSFQ-M-C) was taken home by the patients with PCOS and returned during their first follow-up visit at the Outpatient Clinic of Infertility Treatment. Of 147 women enrolled in our study, 91 provided CSFQ-M-C test results from their partners.

The control group included women without a diagnosis of PCOS or chronic diseases. They provided written consent to the study and completed our demographic questionnaire and the CSFQ-F-C. Their partners met the CSFQ-M-C criteria. All questionnaires were returned together. Of the 93 women included in the control group, 69 took the test.

All information about the physical condition of patients with PCOS came from medical records prepared during hospitalization by doctors of the Department of Gynecological Endocrinology. Information about the control group participants was obtained based on interviews with them.

### 2.1. Questionnaires and Scales

The demographic questionnaire included age, marital status, place of residence, education, employment, confirmation of having a permanent sexual partner, and confirmation of sexual activity in the last week or last month.

The Changes in Sexual Functioning Questionnaire 14 (CSFQ-14) was used to assess sexual dysfunction. The CSFQ-14 is well validated and contains sex-specific items and has proven useful in assessing changes in sexual function in various populations. The questionnaire measures overall sexual functioning (sum of 1 to 14 items), with 5 subdomains assessing pleasure (Item 1), desire/frequency (Items 2 + 3), desire/interest (Items 4 + 5 + 6), arousal (Items 7 + 8 + 9), and orgasm (Items 11 + 12 + 13). We defined sexual dysfunction on a standard basis (CSFQ—female clinical version global score ≤ 41 and CSFQ—male clinical version global score ≤ 47).

The Visual Analogue Scale (VAS) is a psychometric scale that can be used as a tool to measure subjective characteristics or attitudes. In our study, PCOS patients indicated their level of agreement with the question by indicating their position on a solid line between Points 1 and 10. Using the VAS scale used in several previous studies, we obtained 7 questions: (1) How important is a satisfying sex life to you? (2) How many sexual thoughts and fantasies have you had in the past? (3) Do you find yourself sexually attractive? (4) How does excessive body hair affect your sexuality? (5) Does your appearance make it difficult to establish social contacts? (6) In the last 4 weeks, how often have you experienced pain during intercourse? (7) How satisfied have you been with your sex life in the last 4 weeks [11]? The scale was not validated for the Polish version.

The Waist Hip Ratio (WHR) was calculated by dividing a waist measurement by a hip measurement. The Ferriman–Gallwey (m-F-G) score was used to evaluate terminal hair growth on a scale of 0–4 on eleven different body areas according to the authors’ scoring system. A Ferriman–Gallwey score ≥ 8 was considered diagnostic of hirsutism. The presence and severity of acne was evaluated on a scale of 0–4.

### 2.2. Statistical Analyses

A mixed analysis of variance (ANOVA) model was used to examine the differences between women and their partners in PCOS and control groups. In each model, we included within subject effects (i.e., difference between women and their partners), between subject effects (i.e., difference between PCOS and control groups), and interaction between both effects (i.e., to answer the question if the differences between women and men are diverse in PCOS and control groups). We computed the ω^2^ coefficient as a measure of the effect size due to its lower susceptibility in the cases of assumption violations [14]. In the case of statistically significant effects, Holm’s post hoc tests (together with Cohen’s ds as effect size measures) were computed.

To examine the correlation between CSFQ and VAS, we used Pearson’s correlation coefficients. The same procedure was used to explore some demographic and physical correlates of the sexual functioning of women with PCOS. Yet, in this case, having in mind the ordinal (i.e., education) or nominal (i.e., residence, work, acne presence, and hirsutism), we also used Spearman’s correlation (for ordinal variable) and the U Mann–Whitney test (for nominal variables) to explore possible correlates of sexual functioning of women with PCOS.

## 3. Results

### 3.1. Participants

The sample (n = 160) consisted of two subsamples: (1) women diagnosed with PCOS and their partners (n = 91) and (2) women without PCOS and their partners (control group; n = 69). The average age of the women participating in the study was 28.64 years (SD = 5.61) and was slightly higher in the PCOS group (M = 29.61, SD = 6.64) compared to the control group (M = 27, 91, SD = 4.57). However, the difference was not statistically significant (Mann–Whitney U test with rank-biserial correlation as a measure of effect size: W = 3532.00, *p* = 0.176, rbs = 0.09).

Most participants reported higher education (PCOS: n = 51, 56%; control: n = 44, 64%) or secondary education (PCOS: n = 33, 36%; control: n = 24, 35%). The majority also maintained stable work (PCOS: n = 69, 76%; control: n = 61, 88%). However, some of them remained unemployed (PCOS: n = 12, 13%; control: n = 7, 10%) or received a life pension (PCOS: n = 4, 4%; control: n = 1, 1%).

All respondents declared a stable heterosexual relationship. In both subsamples, the percentage of women in a formal relationship (PCOS: n = 42, 46%; control: n = 35, 51%) and an informal relationship (PCOS: n = 49, 54%; control: n = 29, 42%; n = 5, 7% of women in the control group declared they were divorced) remained similar. Most of the respondents lived with their family (PCOS: n = 76, 84%; control: n = 48, 70%; the rest declared living alone).

Regarding the PCOS group, the participants’ average WHR was 0.86 (SD = 0.10), with the lowest value of 0.67 and the highest of 1.17. In the case of 32 (35%) women, the Ferriman–Gallwey test indicated a presence of hirsutism. Also, in the case of 55 (60%) women, the medical examination revealed acne.

### 3.2. Sexual Functioning—Descriptive Statistics

Table 1 presents descriptive statistics for the dimensions of the sexual functioning of women with PCOS and the control group and their partners.

### 3.3. Sexual Functioning—Differences between Women with PCOS, Their Partners, and Women without PCOS

Table 2 presents the differences between women and their partners, PCOS and control groups, and the interaction between both effects.

In terms of pleasure, desire (interest), orgasm, and total score, the analysis showed a statistically significant difference between women and their partners (in all cases: *p* < 0.001), which suggests small (for pleasure) to medium effects (for other variables). However, the difference between PCOS participants and the control group was not statistically significant (pleasure: *p* = 0.971, desire—interest: *p* = 0.668; orgasm: *p* = 0.963; total score: *p* = 0.511). Also, the pattern of differences between women and men was similar in both the PCOS and control groups (pleasure: *p* = 0.877; desire—interest: *p* = 0.101; orgasm: *p* = 0.143; total score: *p* = 0.413), suggesting higher levels pleasure, desire (interest), orgasm and overall sexual functioning reported by men regardless of the presence or absence of a PCOS diagnosis in their partners.

In terms of desire (frequency) and arousal, the analysis showed a statistically significant difference between women and their partners (in all cases: *p* < 0.001), suggesting medium to large effects. However, the difference between PCOS participants and the control group was not statistically significant (desire—frequency: *p* = 0.102; arousal: *p* = 0.938). However, the pattern of discrepancies between women and men differed between the PCOS group and the control group (desire—frequency: *p* = 0.004; arousal: *p* < 0.001), with small effects for all variables. The results suggest that although men generally experienced higher levels of desire (frequency) compared to women (regardless of the presence or absence of a PCOS diagnosis), men in the control group reported higher levels of desire (frequency) compared to women and men who were partners of PCOS participants (mean difference was 0.61; t = 2.81, *p* = 0.016, Cohen’s d = 0.45). In the case of arousal, while women from the control group declared a slightly higher level of arousal compared to women from the PCOS group (the average difference was 0.60; t = 2.04, *p* = 0.085, Cohen’s d = 0.33), men from the control group declared slightly lower arousal compared to men in the PCOS group (mean difference was −0.56; t = −1.91, *p* = 0.085, Cohen’s d = −0.30). Also, the discrepancy in arousal between women and men in the control group was average (the average difference was −1.16; t = −4.91, *p* < 0.001, Cohen’s d = −0.63), while in the PCOS group it was very large (mean difference was −2.32, t = −11.29, *p* < 0.001, Cohen’s d = −1.26).

### 3.4. Sexual Functioning of Women with PCOS—Correlation between CSFQ and VAS

Table 3 presents Pearson’s correlation coefficients between CSFQ and VAS scores.

As expected in Hypothesis 1, the importance (VAS1) and level (VAS7) of sexual satisfaction felt by women diagnosed with PCOS were positively correlated with almost all dimensions of the CSFQ. Therefore, a greater importance of sexual satisfaction was associated with a higher level of pleasure (r = 0.32, *p* = 0.002), frequency of desire (r = 0.24, *p* = 0.025) and interest (r = 0.37, *p* < 0.001), arousal (r = 0.30, *p* = 0.004), and orgasm (r = 0.22, *p* = 0.039), and more satisfying overall sexual functioning (r = 0.35, *p* < 0.001). All correlation coefficients suggested small to medium effects. Accordingly, higher levels of sexual satisfaction were associated with higher levels of pleasure (r = 0.67, *p* < 0.001), frequency of desire (r = 0.48, *p* < 0.001), arousal (r = 0.39, *p* < 0.001), and orgasm (r = 0.53, *p* < 0.001) and more satisfying overall sexual functioning (r = 0.53, *p* < 0.001). All correlation coefficients suggested medium to large effects. Therefore, Hypothesis 1 was confirmed.

Moreover, as expected in Hypothesis 2, the perceived level of personal attractiveness (VAS3) experienced by women diagnosed with PCOS was positively correlated with almost all dimensions of the CSFQ. In contrast, the perceived impact of excess hair on personal sexuality (VAS4) and appearance-related social struggles (VAS5) was negatively associated with only a few CSFQ dimensions. Therefore, a higher level of subjective personal attractiveness was associated with a higher level of pleasure (r = 0.24, *p* = 0.025), desire—interest (r = 0.25, *p* = 0.016), arousal (r = 0.30, *p* = 0.005 ), and orgasm (r = 0.28, *p* = 0.008), and more satisfying overall sexual functioning (r = 0.34, *p* < 0.001). All correlation coefficients suggested small effects. Also, a perceived greater impact of excess hair on personal sexuality (r = −0.26, *p* = 0.013) and greater social struggles due to appearance (r = −0.27, *p* = 0.009) corresponded to lower levels of pleasure. All correlation coefficients suggested small effects. Therefore, Hypothesis 2 was partially confirmed.

As assumed in Hypothesis 3, the intensity of sexual thoughts and fantasies in the last month (VAS2) correlated positively with the dimensions of desire (for frequency: r = 0.46, *p* < 0.001; for interest: r = 0.73, *p* < 0.001). All correlation coefficients suggested medium (for frequency) or large (for percentages) effects. Therefore, Hypothesis 3 was confirmed. Interestingly, sexual fantasies were also positively associated with some other dimensions of the CSFQ, i.e., arousal (r = 0.38, *p* < 0.001) and orgasm (r = 0.25, *p* = 0.016), and with overall sexual functioning (r = 0.52, *p* < 0.001). In all cases, a higher intensity of sexual thoughts and fantasies corresponded to higher levels of desire (frequency and interest), arousal and orgasm, and more satisfying overall sexual functioning.

We also confirmed Hypothesis 4, as the analysis showed a negative correlation between the occurrence of painful sexual intercourse (VAS6) and the orgasm domain (r = −0.27, *p* = 0.011), which suggests that the frequent occurrence of painful sexual intercourse is equally significant—associated with a lower quality of orgasm. Correlation coefficients suggested a small effect. Therefore, Hypothesis 4 was confirmed. Accordingly, higher frequency of painful sexual intercourse was associated with lower levels of several other CSFQ dimensions, i.e., pleasure (r = −0.39, *p* < 0.001) and arousal (r = −0.34, *p* < 0.001), and with lower overall functioning sexual (r = −0.35, *p* < 0.001).

### 3.5. Demographic and Physical Correlates of Sexual Functioning of Women with PCOS

Correlations between age, WHR, Ferriman–Gallwey score, and dimensions of CSFQ were presented in Table 4. As other demographic variables were either ordinal (i.e., education) or nominal (i.e., residence, acne presence, and hirsutism), we completed the analyses with Spearman’s correlation (for education—also presented in Table 4) and the U Mann–Whitney test (Table 5).

Correlation analyses showed that neither the WHR score nor the Ferriman–Gallwey score were associated with CSFQ dimensions and overall sexual functioning in women with PCOS. Also, the level of education was not statistically significant in relation to sexual functioning. However, the analyses showed that the older the women were, the higher the quality of orgasm they experienced (r = 0.21; *p* = 0.041). The correlation coefficient suggested a small effect.

The results of the U Mann–Whitney test did not reveal statistically significant differences between women diagnosed with PCOS: (1) with present and absent acne, (2) with and without hirsutism. The only difference was reported with respect to residence. In this case, women with PCOS who lived alone reported a higher level of desire (interest; *p* = 0.030) compared to those who lived with their families (for women who lived alone: median = 10.0, IQR = 3,50; for women who lived with their families: median = 8.50, IQR = 3.00).

### 3.6. Associations between VAS Scores and Physical Features (WHR, Acne, and Hirsutism)

To analyze the associations between VAS scores and some physical features (i.e., WHR and Ferriman–Gallwey score), we perform Pearson’s r correlation (Table 6).

The results showed statistically significant positive correlations between the WHR ratio, social struggles related to appearance (VAS5; r = 0.23, *p* = 0.031), and level of last month’s sexual satisfaction (VAS7; r = 0.30, *p* = 0.004). In both cases, the higher the WHR was, the higher both the social struggles related to appearance and the declared sexual satisfaction were. Both statistical effects were small. 

Also, the Ferriman–Gallwey score was positively correlated with the level of perceived impact of excessive hair on personal sexuality (VAS4; r = 0.41, *p* < 0.001). It suggested that women diagnosed with PCOS with higher Ferriman–Gallwey scores declared higher levels of perceived impact of excessive hair on personal sexuality. In this case, the effect size was medium.

To analyze the associations between VAS scores and other physical characteristics (i.e., presence of hirsutism and acne), we performed a Mann–Whitney U test, with the same rationale as in the previous paragraph.

Overall, the results showed no statistically significant differences in VAS scores between groups of women diagnosed with PCOS with and without acne. The only difference that turned out to be statistically significant concerned the number of painful sexual contacts (VAS6), which was higher in women with PCOS and acne (median = 2.00, IQR = 4.00) compared to women without acne (median = 1.00, IQR = 1.00). However, the effect size of this difference was rather small. As a result, the results showed no statistically significant differences in VAS results between groups of women diagnosed with PCOS with and without hirsutism. In this case, the only difference that turned out to be statistically significant was the perceived impact of excessive hair on personal sexuality (VAS4), which was higher in women with PCOS and hirsutism (median = 7.00, IQR = 6.00) compared to people without hirsutism (median = 2.00, IQR = 4.00). The effect size of this difference was medium.

### 3.7. Association between Sexual Activity in Last Week and Sexual Functioning in Women Diagnosed with PCOS

We examined the association between sexual activity and sexual functioning in women diagnosed with PCOS using the Mann–Whitney U test. We decided on this procedure due to (1) the dichotomous nature of the variable related to sexual activity (possible answers: yes or no) and (2) differences in n groups (in the case of sexual activity in the last week—for “no”: n = 56; for “yes”: n = 35). Also, taking into account a small n (i.e. the variable indicating sexual activity in the last month—for “no”: n = 8; for “yes”: n = 83) or the lack of cases in one group (i.e. the variable indicating sexual activity during the last three months) in the case of other variables related to sexual activity, we decided to conduct this test only for the variable related to sexual activity during the last week. 

The results showed no statistically significant differences in sexual functioning between the group of women with PCOS who declared sexual activity within the last week and those who did not perform any sexual activity in that time.

## 4. Discussion

The impact of PCOS on sexual function in women is still a matter of debate. Although reviews and meta-analyses devoted to the topic of sexual dysfunction in women with PCOS [11,12,13] do not lead to one consistent conclusion, they do indicate that there is a possible relationship between PCOS and FSD in these women. Pastor et al. showed that women with PCOS experienced FSD in terms of arousal, lubrication, sexual satisfaction, orgasm, and overall scores compared to controls [11]. However, Zhao et al. showed that there are no statistically significant differences in FSD between women with PCOS and women without this diagnosis [12]. Loh et al. conducted the most extensive meta-analysis and found that women with PCOS have a 30% higher risk of developing FSD compared to women without PCOS [13]. They confirmed that total scores on the Female Sexual Function Index (FSFI) did not differ significantly between the study and control groups, while women with PCOS obtained significantly lower scores on the pain and satisfaction subscales than the control groups [13]. In their opinion, this indicated limitations in the use of this scale to assess sexual desire [13] and the overlap between desire and arousal [15]. Regardless of the limitations of FSFI, it is important to remember that both men and women may have difficulty distinguishing between desire and arousal because sexual stimuli trigger both desire and arousal simultaneously [16,17].

Stovall et al. are the only ones in the past who have used the CSFQ questionnaire to assess sexual functions in women with PCOS [18]. Although they described decreased sexual function in most domains, they showed statistically significant differences between the study and control groups only in the orgasm/fulfillment domain. In our study, we did not obtain statistically significant differences between women with PCOS and the control group in terms of domains and the total score on the CSFQ scale confirming the occurrence of FSD in the study group. However, a detailed analysis of the CSFQ subscales showed that in both groups of women the average values of the subscales of pleasure (3.92 vs. 3.93), desire/interest (8.75 vs. 8.58) and arousal (10.56 vs. 11, 16) were below the cut-off limit—points excluded (Table 1). Additionally, the mean values in the orgasm domain (10.88) were also below the threshold for sexual dysfunction in women with PCOS (Table 1). The results presented are like those reported by Stovall et al. and generally support mild FSD in women; we would like to note, however, that our study provides insight into the sexuality of women with PCOS in the context of their sexual partners. In terms of all domains and general functioning on the CSFQ scale, we obtained a pattern of statistically significant differences between women and men in favor of men (Table 2). This effect ranged from small to medium on the pleasure, desire/interest, orgasm, and total score subscales, but from medium to large on the desire/frequency and arousal subscales.

To explain the above results, it is necessary to refer to sexual response models. The linear model supplemented by Kaplan included four phases: desire, arousal, orgasm, and decisiveness, and assumed that male desire is stable and high [19]. The latest research focuses on assessing the short-term variability of the desires of women and men in the context of their emotional state and closeness in a partnership and does not confirm significant differences between women and men [20]. This is consistent with Basson’s later model assuming that the motivations for sexual activity are numerous, sexual desire does not have to be present at the beginning of the sexual response, but it is achieved after the brain processes sexual signals that combine desire with arousal [21]. It has been suggested that women with different levels of sexual function identify with different models of the sexual response cycle [22], and women identifying with Basson’s model had significantly lower levels of sexual function [23]. Clinical observations confirm that the dominant reasons for initiating and partnering in sexual activity in women are emotional, and in men, physical [21]. The central role of attention processes in stimulating the subjective and physiological components of sexual arousal has been proven [24]. Conscious evaluation of sexual stimuli and contextual cues can lead to subjective sexual arousal (SSA). The latter may be increased by awareness of genital congestion, which is more typical of the male experience [25].

(SSA) has been defined as positive cognitive [26] or emotional engagement [27] in response to a sexual stimulus, suggesting that one must be directly or indirectly aware of the sexual stimulus, which may be internal (sexual thoughts) or external (partner’s), to experience SSA [28]. SSA reduces sexual restraint [29] and motivates individuals to engage in sexual activity [30]. It is also believed that increased SSA may increase pleasure and satisfaction during sexual activity, which are associated with engagement in future sexual activity [31]. This knowledge may lead us to another result in our study, that the intensity of sexual thoughts and fantasies was associated with higher levels of desire/frequency, desire/interest, but also arousal and orgasm (Table 3). Attempts to enhance SSA by administering drugs did not produce consistent results, which is most likely related to the excessive heterogeneity of the studied women and the drug dose [32,33,34]. Cognitive interventions are more effective (91% of studies) than pharmacological ones (31% of studies) [35]. This may be partially explained by psychological variables (mood, attention, relationship satisfaction), which may have a stronger impact on women’s experience of SSA than pharmacological agents [35]. Velten et al. showed that women with clinical and subclinical sexual dysfunctions engage in sexual stimuli to a lesser extent [36]. This finding suggests that therapeutic efforts should focus on increasing attention to sexual cues and simultaneously analyzing women’s emotional and cognitive evaluation of these cues [36]. 

Empirical research indicates that the motivation to engage in sexual activity is related to sexual thoughts and fantasies, which influence the level of sexual well-being [21]. Gender differences in the way women and men respond to sexual situations highlight the importance of the potential for sexual arousal to decline or disappear “if everything goes wrong” [21]. In this context, the most interesting result concerns the domain of arousal in women with PCOS. They declare only slightly lower arousal compared to women from the control group, but there is a large discrepancy between them and their partners in this respect (Table 2). This may be complemented by the dual control model, which states that sexual arousal is influenced by excitatory and inhibitory mechanisms, with women generally exhibiting greater inhibition and men greater arousal [37,38]. The central neuroendocrine mechanisms regulating the sexual response in women are today described as dynamic, creating a balance between stimulating and inhibiting factors [39,40]. Several studies have confirmed the occurrence of arousal disorders in women with PCOS [12,41,42,43,44]. Bazarganipour et al. noticed a possible relationship between arousal and hirsutism, acne, and obesity [42]. Bahadori et al. showed that arousal dysfunction was associated with phenotype B characterized by hyperandrogenism, hirsutism, and anovulation [43]. Gniew et al. suggested that this may be due to the degree of personal distress observed within the team [44]. In this context, our results require further research due to the lack of an objective assessment of the affective state and the relationship with specific phenotypic characteristics of PCOS patients.

The assessment of individual sexual domains leads to the conclusion that women with PCOS derive significantly less satisfaction from their sexual life than women without this syndrome [11,41,45,46]. It may be the result of specific symptoms such as hyperandrogenism, hirsutism, and oligoanovulation or anovulation, characteristic of both phenotype A and B [47]. These symptoms had a negative impact on physical appearance, resulting in a decreased perception of “feminine identity” and a feeling of “unattractiveness” [48], as well as a deterioration of “self-image” and lowered “self-esteem” [49]. Indeed, higher levels of subjective attractiveness (VAS3) in our patients were associated with higher scores in almost all domains and the CSFQ total score, except for the desire/frequency subscale (Table 3). This finding indicates the importance of a positive attitude towards oneself, which can strengthen individual psychological mechanisms that eliminate frustration caused by the diagnosis and course of PCOS.

Eftenkhar et al. found lower scores in all FSFI domains if the Ferriman–Gallwey scale scores were 6–8 [50]. Elsenbruch et al. described concerns about excessive hair growth among women with PCOS, who confirmed that it had a detrimental effect on their sexual life [45]. Our study shows two conclusions on this subject: firstly, the greater the impact of excessive hair on sexuality (VAS4) and the greater social problems resulting from appearance (VAS5), the lower the level of pleasure was (Table 3), and secondly, the higher results on the Ferriman–Gallwey scale worsened the subjectively perceived impact of excessive hair on sexuality (VAS4) (Table 6). Ercan et al. reported a significant negative correlation between the results of total FSFI and the levels of total and free testosterone [51]. Veras et al. showed a negative correlation between sexual functions and the levels of total testosterone and dehydroepiandrosterone sulfate [52]. On the contrary, Mansson et al. and Stovall et al. proved the inverse relationship [18,41]. Rellini et al. confirmed that clinical symptoms suggesting sensitivity to androgen levels, but not biological androgen levels per se, predicted levels of sexual desire [53]. In this context, the role of testosterone in modulating female sexual desire and sexual function in general is still poorly understood. Although there is evidence of benefits from short-term transdermal testosterone administration in postmenopausal women with low sexual drive, there is no specific serum level or lower limit for androgens or androgen precursors to recognize decreased female sexual function [54,55]. Our findings confirm that physical characteristics associated with hyperandrogenism have a negative impact on women’s sexuality.

There are various research results describing the association of sexual functions with obesity, BMI, and WHR in women with PCOS, namely that obesity and hirsutism correlate with reduced sexual satisfaction [56], an increase in BMI has a negative relationship with satisfaction with sexual life [41], with lower results in FSFI [57], an increase in body weight and abdominal circumference negatively correlated with sexual satisfaction in women [58,59], and a progressive increase in sexual dysfunctions correlated with a constant increase in the WHR index [60]. De Frene et al. concluded that an increase in BMI in women leads to a decrease in sexual satisfaction and in their relationships, but this was inconsistent with the feelings of their partners, which increased in proportion to their body weight [61]. Benetti-Pinto et al. noticed that BMI correlates with quality of life, i.e., the higher the BMI, the lower the quality of life [62]. The studies by Elsenbruch et al. and Stovall et al. showed that BMI changes did not correlate with the level of satisfaction in women with PCOS [18,45]. In the case of our study, it was shown that the higher the WHR index, the greater the social problems resulting from appearance and the greater the sexual satisfaction reported by women with PCOS in the last month (Table 6). It cannot be concluded that a higher WHR had a negative impact on perceived sexual satisfaction, but it did cause greater social struggles.

The more frequent occurrence of painful sexual intercourse in our patients is associated with a lower quality of orgasm, as well as a lower level of pleasure, arousal, and overall functioning (Table 3). Loh et al. showed a lower pain score in women with PCOS, which they interpreted as the possibility of dyspareunia caused by relatively low FSH levels preventing the growth of ovarian follicles and resulting in estrogen deficiency with possible vaginal atrophy [13]. It is worth mentioning the results of the study by Nohr EA et al., who found that dyspareunia occurs more often in the group of patients with a history of PCOS [63]. The authors interpreted this finding in the context of decreased thirst and reduced ability to relax due to chronic pain and medical conditions. This observation is limited by the fact that the mean age of the patients was over 40 years. In this context, our results call for deeper research into the somatic causes of intercourse pain.

Research on sexual functions poses many methodological difficulties, which are also visible in our study. The described discrepancy in results—the presence vs. absence of FSD and statistical difference vs. absence of this difference compared to the control group—can be explained by the multifactorial nature of women’s sexual functions, the severity of PCOS, and the nature of sexual relationships, as well as different populations of research groups, inclusion criteria, and assessment methods [64]. Additionally, the limited number of participants in the study group did not allow for the analysis of sexual functions in terms of specific PCOS phenotypes and sex hormone levels. 

## 5. Conclusions

We confirmed that there are no statistically significant differences in sexual function between women with PCOS and women without this diagnosis. This finding concerns young cisgender women who reported living in stable heterosexual relationships. Our attempt to compare the sexual function of women and their partners in the study group showed significant differences in all sexual areas and total sexual functioning, but the difference in arousal was particularly important. The intensity of sexual thoughts and fantasies especially increased desire. We have proven that self-esteem has a huge impact on women’s sexuality. Our results concern young cisgender women who reported living in stable heterosexual relationships.

Our findings should be translated into a model of care for women with PCOS. Multidisciplinary care models should offer integrated care with tailored therapies, education, and lifestyle support, as well as treatment. A wide range of health care for this group of patients should include not only gynecologists, endocrinologists, primary care physicians, dietitians, and exercise physiologists, but open access to psychologists and sexologists should be ensured. The involvement of various specialists should facilitate the preparation of a question list, a structured list of patient-generated and applied health questions that can be integrated with evidence-based information and answers. This facilitates patient–caregiver interaction and may contribute to improving patient knowledge and support [1].

## Figures and Tables

**Table 1:** Sexual functioning of women with and without PCOS and their partners—descriptive statistics.

	*M*	*SD*	Min	Max	Skewness
PCOS group—women					
Pleasure	3.92	0.96	1.00	5.00	−0.78
Desire—frequency	6.95	1.37	3.00	10.00	−0.35
Desire—interest	8.75	2.06	3.00	12.00	−0.34
Arousal	10.56	2.05	5.00	14.00	−0.37
Orgasm	10.88	2.46	3.00	15.00	−0.40
Total CSFQ	49.59	7.47	27.00	63.00	−0.34
Control group—women					
Pleasure	3.93	0.99	1.00	5.00	−0.98
Desire—frequency	6.97	1.40	4.00	10.00	−0.28
Desire—interest	8.58	2.45	3.00	13.00	−0.29
Arousal	11.16	2.10	6.00	15.00	−0.83
Orgasm	11.15	2.94	3.00	15.00	−1.02
Total CSFQ	50.68	8.63	24.00	64.00	−1.31
PCOS group—men					
Pleasure	4.28	0.82	1.00	5.00	−1.55
Desire—frequency	7.42	1.39	4.00	10.00	−0.18
Desire—interest	9.77	2.19	4.00	15.00	−0.01
Arousal	12.88	1.46	9.00	15.00	−0.84
Orgasm	12.40	1.67	7.00	15.00	−0.71
Total CSFQ	56.12	5.55	40.00	70.00	−0.51
Control group—men					
Pleasure	4.26	0.82	1.00	5.00	−1.36
Desire—frequency	8.03	1.28	4.00	10.00	−0.31
Desire—interest	10.19	1.84	6.00	15.00	0.02
Arousal	12.32	1.72	8.00	15.00	−0.44
Orgasm	12.10	1.87	7.00	15.00	−0.68
Total CSFQ	56.33	6.37	37.00	70.00	−0.74

Scoring for CSFQ-F-C (a score at or below the cut-off points indicative for sexual dysfunction) Total CSFQ: 41.0, Pleasure: 4.0, Desire/frequency: 6.0, Desire/interest: 9.0, Arousal: 12.0, Orgasm: 11.0. Scoring for CSFQ-M-C (a score at or below the cut-off points indicative for sexual dysfunction) Total CSFQ: 47.0, Pleasure: 4.0, Desire/frequency: 8.0, Desire/interest: 11.0, Arousal: 13.0, Orgasm: 13.0.

**Table 2:** Differences between women with PCOS, their partners, and women without PCOS—results of mixed ANOVA.

	Effect	*F*	*df*	*p*	ω^2^
Pleasure	Within subject	33.80	1, 158	<0.001	0.03
	Between subject	0.00	1, 158	0.971	0.00
	Within × between subject	0.02	1, 158	0.877	0.00
Desire—frequency	Within subject	59.42	1, 158	<0.001	0.07
	Between subject	2.70	1, 158	0.102	0.01
	Within × between subject	8.69	1, 158	0.004	0.01
Desire—interest	Within subject	54.71	1, 158	<0.001	0.08
	Between subject	0.19	1, 158	0.668	0.00
	Within × between subject	2.72	1, 158	0.101	0.00
Arousal	Within subject	123.57	1, 158	<0.001	0.18
	Between subject	0.01	1, 158	0.938	0.00
	Within × between subject	13.73	1, 158	<0.001	0.02
Orgasm	Within subject	42.25	1, 158	<0.001	0.07
	Between subject	0.00	1, 158	0.963	0.00
	Within × between subject	2.17	1, 158	0.143	0.00
Total CSFQ	Within subject	130.35	1, 158	<0.001	0.16
	Between subject	0.43	1, 158	0.511	0.00
	Within × between subject	0.67	1, 158	0.413	0.00

**Table 3:** Sexual functioning of woman with PCOS—Pearson’s correlation coefficients between CSFQ and VAS scores.

	1	2	3	4	5	6	7	8	9	10	11	12
1. Pleasure	—											
2. Desire—frequency	0.44 ***	—										
3. Desire—interest	0.16	0.55 ***	—									
4. Arousal	0.64 ***	0.55 ***	0.49 ***	—								
5. Orgasm	0.58 ***	0.37 ***	0.35 ***	0.63 ***	—							
6. Total CSFQ	0.69 ***	0.70 ***	0.66 ***	0.89 ***	0.80 ***	—						
7. Sexual satisfaction importance (VAS1)	0.32 **	0.24 *	0.37 ***	0.30 **	0.22 *	0.35 ***	—					
8. Sexual thoughts and fantasies—last month (VAS2)	0.20	0.46 ***	0.73 ***	0.38 ***	0.25 *	0.52 ***	0.55 ***	—				
9. Personal sexual attractiveness (VAS3)	0.24 *	0.20	0.25 *	0.30 **	0.28 **	0.34 ***	0.26 *	0.27 *	—			
10. Impact of excessive hair on personal sexuality (VAS4)	−0.26 *	−0.18	−0.06	−0.11	−0.19	−0.19	0.01	0.06	−0.16	—		
11. Social struggles due to appearance (VAS5)	−0.27 **	−0.11	0.08	−0.11	−0.19	−0.17	0.07	0.22 *	−0.18	0.49 ***	—	
12. Painful sexual encounters (VAS6)	−0.39 ***	−0.13	0.05	−0.34 ***	−0.27 *	−0.35 ***	−0.02	0.03	−0.06	0.10	0.21 *	—
13. Level of sexual satisfaction—last month (VAS7)	0.67 ***	0.48 ***	0.14	0.39 ***	0.53 ***	0.53 ***	0.30 **	0.27 **	0.18	−0.18	−0.04	−0.14

* *p* < 0.05; ** *p* < 0.01; *** *p* < 0.001.

**Table 4:** Demographic and physical correlates of sexual functioning of women with PCOS—Pearson’s correlations between age, WHR, Ferriman–Gallwey score, and dimensions of the CSFQ.

	Pleasure	Desire—Frequency	Desire—Interest	Arousal	Orgasm	Total
Age	0.01	−0.06	−0.07	0.08	0.21 *	0.09
WHR	0.07	0.02	−0.17	−0.05	0.05	−0.03
Ferriman–Gallwey score	0.03	0.07	−0.09	−0.03	−0.16	−0.07
Education	−0.03	0.02	0.13	0.02	−0.05	0.05

As the correlation matrix for CSRQ dimensions for PCOS women was presented in Table 3, we omitted this part of the current table. For education, Spearman’s correlation coefficient was calculated. * *p* < 0.05.

**Table 5:** Demographic and physical correlates of sexual functioning of women with PCOS—U Mann–Whitney test results (grouping variables: residence, acne presence, and hirsutism).

		*W*	*p*	*r_bs_*
Grouping variable: Residence (alone vs. with family)	Pleasure	64.50	0.429	0.12
	Desire—frequency	72.50	0.099	0.26
	Desire—interest	77.50	0.030	0.35
	Arousal	675.50	0.255	0.19
	Orgasm	507.00	0.500	−0.11
	Total CSFQ	668.00	0.296	0.17
Grouping variable: Acne presence (yes vs. no)	Pleasure	921.50	0.560	−0.07
	Desire—frequency	866.00	0.303	−0.13
	Desire—interest	892.50	0.426	−0.10
	Arousal	926.50	0.604	−0.06
	Orgasm	947.50	0.731	−0.04
	Total CSFQ	921.00	0.578	−0.07
Grouping variable: Hirsutism (yes vs. no)	Pleasure	918.00	0.823	−0.03
	Desire—frequency	858.00	0.466	−0.09
	Desire—interest	958.50	0.906	0.02
	Arousal	853.00	0.446	−0.10
	Orgasm	92.50	0.847	−0.03
	Total CSFQ	928.50	0.901	−0.02

For effect size, rank-biserial (*r_bs_*) correlation was reported.

**Table 6:** Associations between VAS scores and physical features (WHR and Ferriman–Gallwey score)—Pearson’s r correlation coefficients.

	1	2	3	4	5	6	7	8
1. WHR ratio	—							
2. Ferriman–Gallwey score	0.19	—						
3. Sexual satisfaction importance (VAS1)	−0.02	0.12	—					
4. Sexual thoughts and fantasies—last month (VAS2)	−0.09	−0.03	0.55 ***	—				
5. Personal sexual attractiveness (VAS3)	−0.18	0.03	0.26 *	0.27 *	—			
6. Impact of excessive hair on personal sexuality (VAS4)	0.07	0.41 ***	0.01	0.06	−0.16	—		
7. Social struggles due to appearance (VAS5)	0.23 *	0.15	0.07	0.22 *	−0.18	0.49 ***	—	
8. Painful sexual encounters (VAS6)	−0.03	−0.07	−0.02	0.03	−0.06	0.10	0.21 *	—
9. Level of sexual satisfaction—last month (VAS7)	0.30 **	0.05	0.30 **	0.27 **	0.18	−0.18	−0.04	−0.14

* *p* < 0.05; ** *p* < 0.01; *** *p* < 0.001.

## Data Availability

Patient medical records are available in patient medical records and are available upon request from the authors: Paweł Madej and Marta Kochanowicz.
